# Supplementary material for: Mixtures of strategies underlie rodent behavior during reversal learning
Source: PLoS Comput Biol. 2023 Sep 14;19(9):e1011430. doi: 10.1371/journal.pcbi.1011430 (PMC10501641; doi:10.1371/journal.pcbi.1011430)
Supplement: S1 Fig — (a) Quantification of late performance of all mice across all sessions (mean ± s.e.m, n = 21 mice). Late performance was calculated by averaging each mouse’s performance in the last 10 trials of each block on each session. (b) Quantification of number of initial errors for all mice across all sessions (mean ± s.e.m, n = 21 mice). Dashed lines in (a) and (b) indicate optimal win-stay-lose-switch strategy (WSLS). WSLS strategy should yield 100% late performance, and should only incur 1 initial error per block. (c) Individual animal average performance on the last 5 sessions (shown on y-axis) and average left–right bias on the last 5 sessions (shown on x-axis). Each point represents one animal with the annotated ID. Points are colored by the number of block HMM modes of each animal (Figs 4E and 6A). (d) Difference in performance between left and right blocks (mean ± standard deviation) across all animals on each training session. (e) Performance across training sessions (mean ± standard deviation) for male (n = 9; black) and female mice (n = 12; blue). (f) Performance of male and female mice (mean ± standard deviation) across three session groups: sessions 1–10, sessions 11–20, and sessions 21–30. (DOCX) [file pcbi.1011430.s001.docx]

**
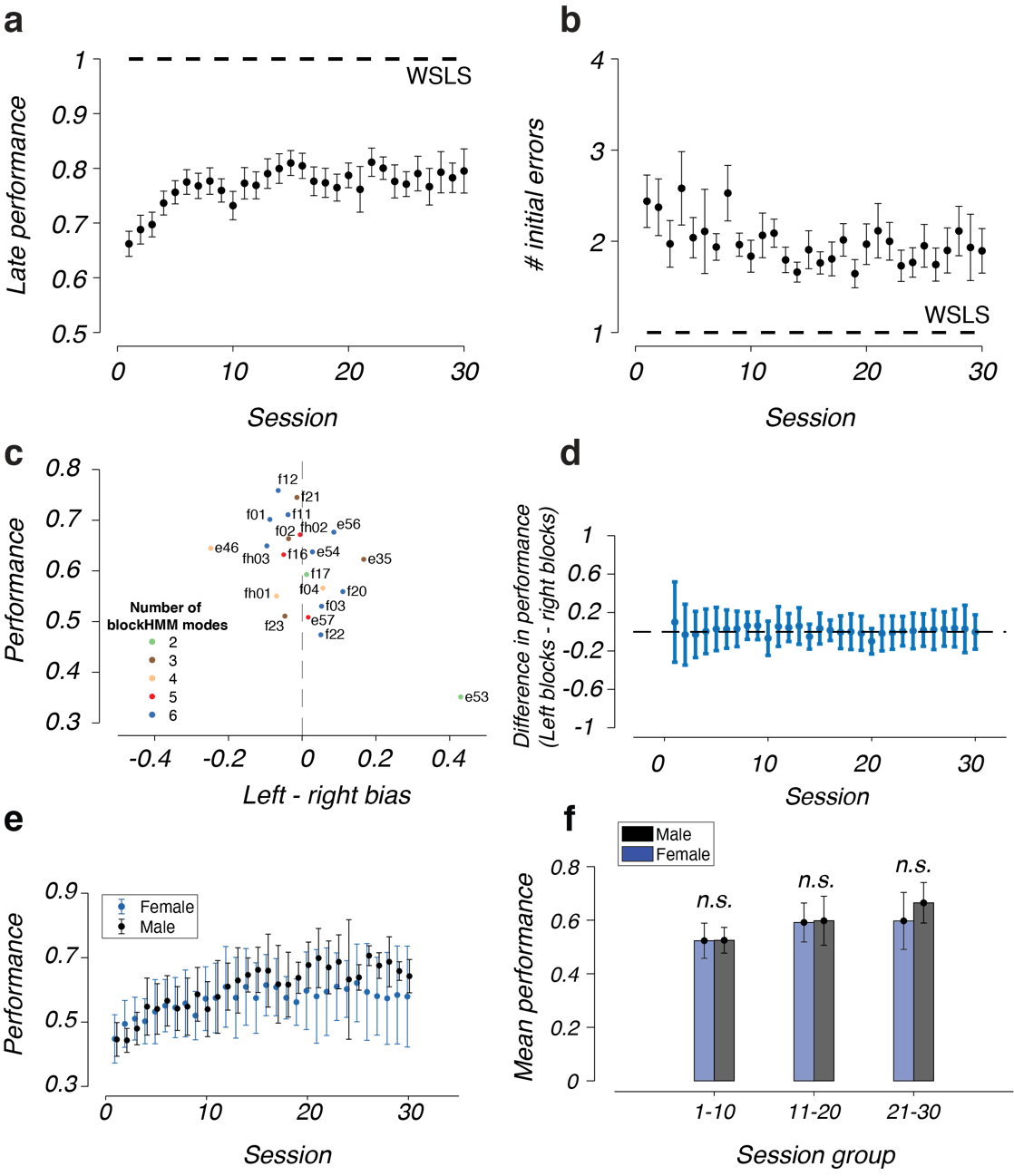
**

**S1 Fig: Quantification of sources of the sub-optimal performance compared to the win-stay-lose-switch strategy.** (a) Quantification of late performance of all mice across all sessions (mean ± s.e.m, *n* = 21 mice). Late performance was calculated by averaging each mouse's performance in the last 10 trials of each block on each session. (b) Quantification of number of initial errors for all mice across all sessions (mean ± s.e.m, *n* = 21 mice). Dashed lines in (a) and (b) indicate optimal win-stay-lose-switch strategy (WSLS). WSLS strategy should yield 100% late performance, and should only incur 1 initial error per block. (c) Individual animal average performance on the last 5 sessions (shown on y-axis) and average left – right bias on the last 5 sessions (shown on x-axis). Each point represents one animal with the annotated ID. Points are colored by the number of block HMM modes of each animal (Figs 4E and 6A). (d) Difference in performance between left and right blocks (mean ± standard deviation) across all animals on each training session. (e) Performance across training sessions (mean ± standard deviation) for male (*n* = 9; black) and female mice (*n* = 12; blue). (f) Performance of male and female mice (mean ± standard deviation) across three session groups: sessions 1-10, sessions 11-20, and sessions 21-30.
