# Supplementary material for: Mixtures of strategies underlie rodent behavior during reversal learning
Source: PLoS Comput Biol. 2023 Sep 14;19(9):e1011430. doi: 10.1371/journal.pcbi.1011430 (PMC10501641; doi:10.1371/journal.pcbi.1011430)
Supplement: S2 Fig — (a) Selection of the number of modes, K, used for blockHMM fitting. Each panel shows the normalized cross-validated log-likelihood for different values of K ranging from 1 to 6. The value of K that maximized the cross-validated log-likelihood is indicated by the vertical dashed line. (b) Distribution of the number of blockHMM modes in male and female mice. (c) Composition of performance-based behavioral regimes in male and female mice. Both female and male mice used four behavioral regimes during their learning. (DOCX) [file pcbi.1011430.s002.docx]

**
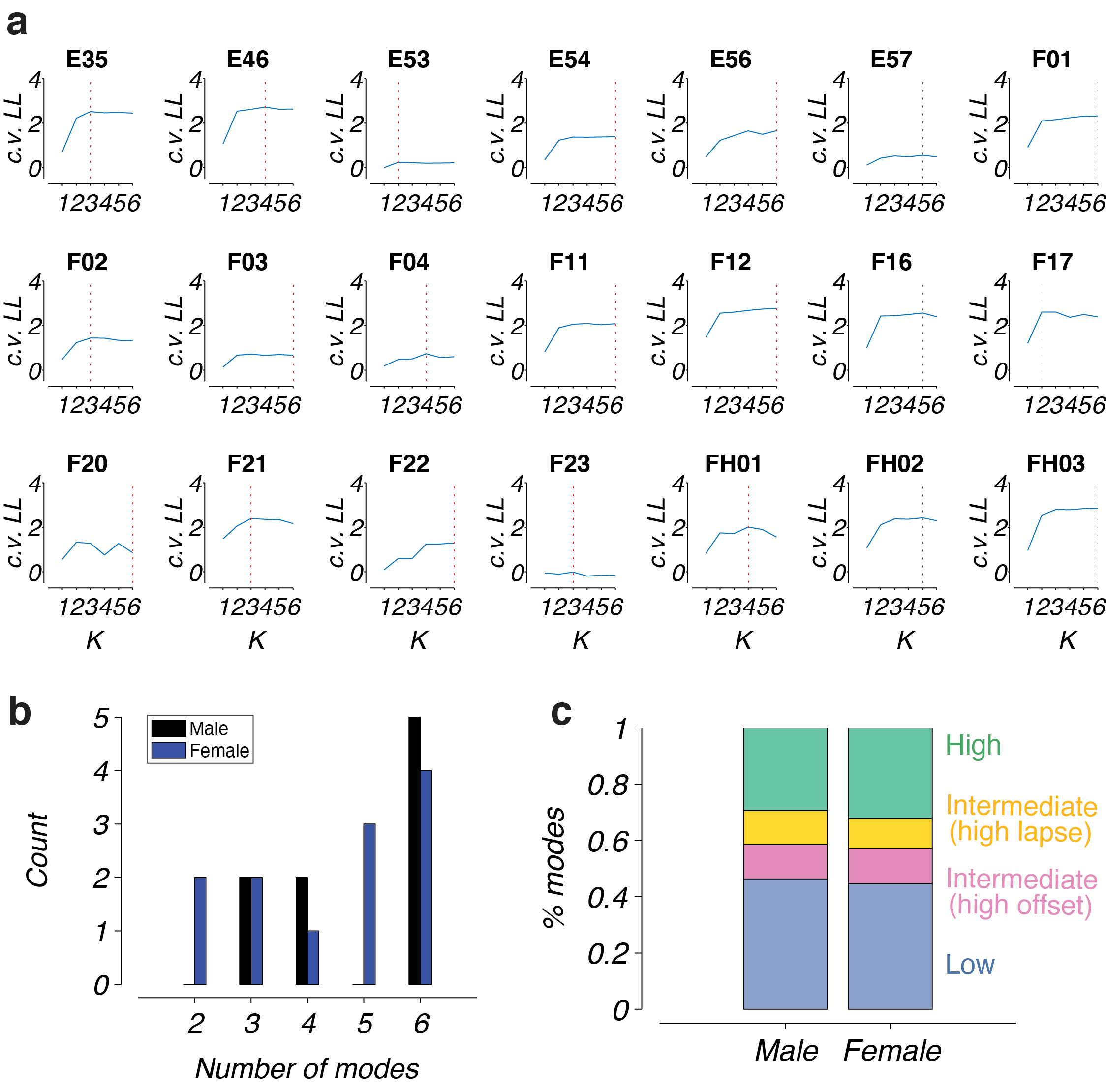
**

**S2 Fig: Quantification of sex differences in the HMM modes and performance-based behavioral regimes.** (a) Selection of the number of modes, *K*, used for blockHMM fitting. Each panel shows the normalized cross-validated log-likelihood for different values of *K* ranging from 1 to 6. The value of *K* that maximized the cross-validated log-likelihood is indicated by the vertical dashed line. (b) Distribution of the number of blockHMM modes in male and female mice. (c) Composition of performance-based behavioral regimes in male and female mice. Both female and male mice used four behavioral regimes during their learning.
