# Supplementary material for: Mixtures of strategies underlie rodent behavior during reversal learning
Source: PLoS Comput Biol. 2023 Sep 14;19(9):e1011430. doi: 10.1371/journal.pcbi.1011430 (PMC10501641; doi:10.1371/journal.pcbi.1011430)
Supplement: S3 Fig — In each matrix, entry Mij shows the transition probability between states P(zt = j | zt-1 = i). Each state is labeled with the decoded behavioral strategy (Q1-Q4 or IB5-6) shown in Fig 6A. (DOCX) [file pcbi.1011430.s003.docx]

**
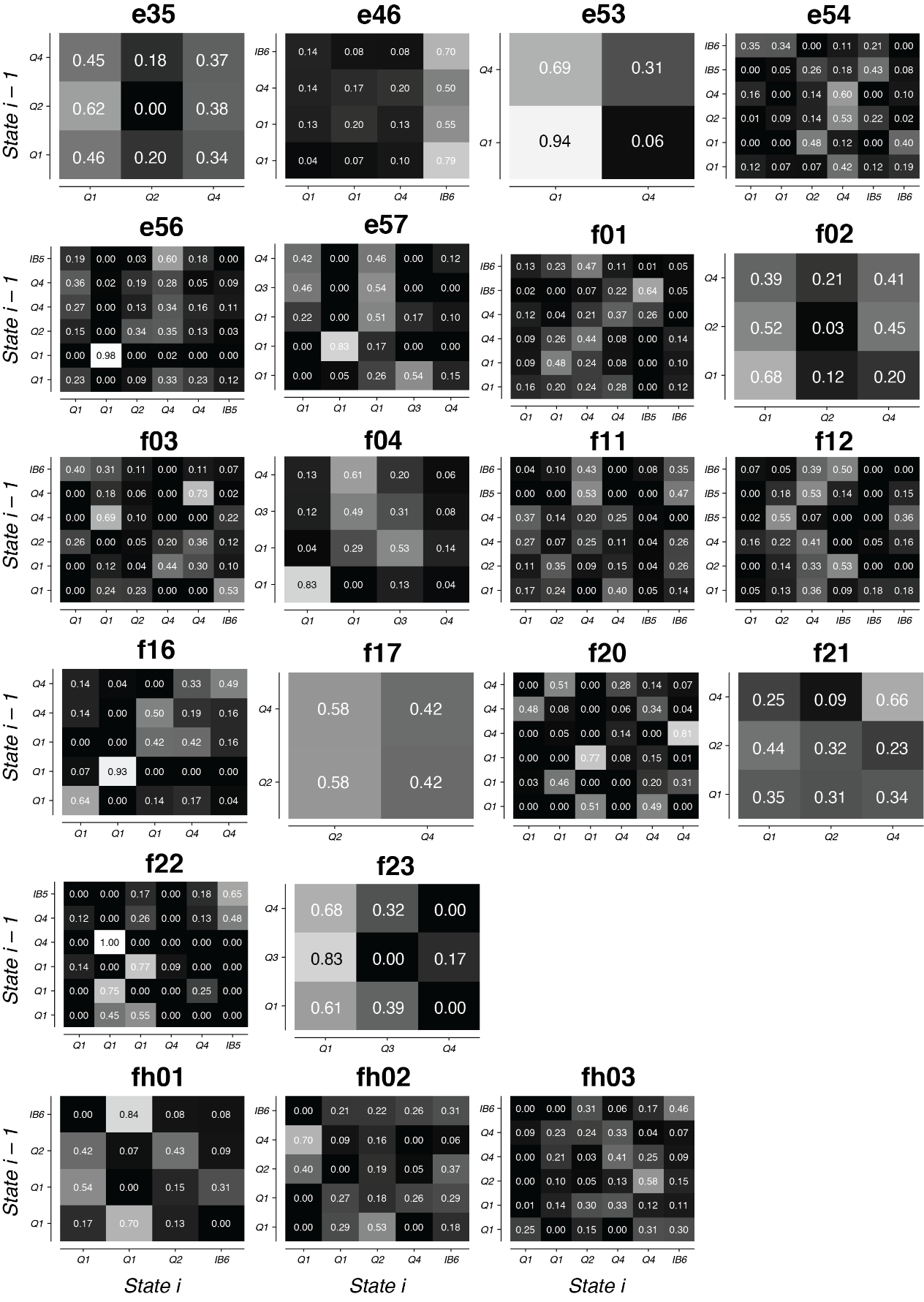
**

**S3 Fig: BlockHMM transition matrices for individual experimental animals.** In each matrix, entry *M_ij_* shows the transition probability between states *P*(*z_t_* = *j* | *z_t_*_-1_ = *i*). Each state is labeled with the decoded behavioral strategy (Q1-Q4 or IB5-6) shown in Fig 6A.
