# Supplementary material for: Mixtures of strategies underlie rodent behavior during reversal learning
Source: PLoS Comput Biol. 2023 Sep 14;19(9):e1011430. doi: 10.1371/journal.pcbi.1011430 (PMC10501641; doi:10.1371/journal.pcbi.1011430)
Supplement: S4 Fig — a) Illustration of the sigmoidal transition function with four parameters: switch offset s, switch slope α, lapse ε, and overall foraging efficiency E. b) Behavioral features for Q-learning agents in a 100–0 environment. We simulated the behavior of 25 x 20 Q-learning agents with different values of the learning rate γ and exploration parameter ε, and measured the four behavioral features for each agent by fitting the average transition function over 1000 blocks to a sigmoidal function. c) Example behavior of three Q-learning agents with a fixed ∊ = 0.1 and varying learning rate γ. Top row shows the behavior of each agent over 100 blocks (each row represents the outcomes of all the trials within a single block, red: incorrect choice, blue: correct choice). Bottom row shows the average transition function (black curve, mean ± standard deviation, n = 1000 blocks), and the fitted sigmoid (blue curve). d) Same as c, but for three Q-learning agents with fixed γ = 1.2 and varying ∊. (DOCX) [file pcbi.1011430.s004.docx]

**
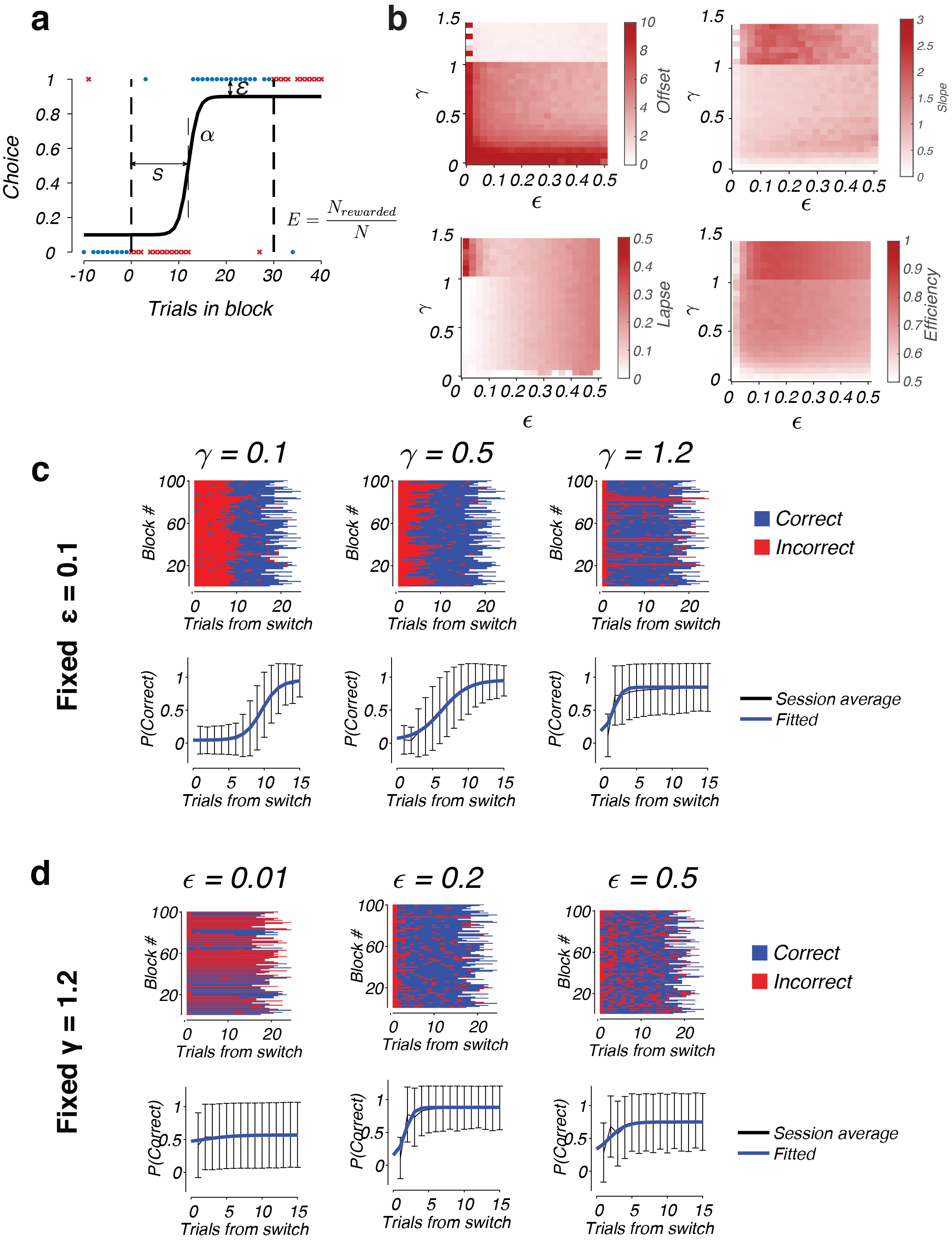
**

**S4 Fig: Variations of four key behavioral benchmarks across the Q-learning parameter space**. a) Illustration of the sigmoidal transition function with four parameters: switch offset s, switch slope α, lapse ε, and overall foraging efficiency E. b) Behavioral features for Q-learning agents in a 100-0 environment. We simulated the behavior of 25 x 20 Q-learning agents with different values of the learning rate γ and exploration parameter ε, and measured the four behavioral features for each agent by fitting the average transition function over 1000 blocks to a sigmoidal function. c) Example behavior of three Q-learning agents with a fixed 𝜖 = 0.1 and varying learning rate 𝛾. Top row shows the behavior of each agent over 100 blocks (each row represents the outcomes of all the trials within a single block, red: incorrect choice, blue: correct choice). Bottom row shows the average transition function (black curve, mean ± standard deviation, n = 1000 blocks), and the fitted sigmoid (blue curve). d) Same as c, but for three Q-learning agents with fixed 𝛾 = 1.2 and varying 𝜖.
