# Supplementary material for: Mixtures of strategies underlie rodent behavior during reversal learning
Source: PLoS Comput Biol. 2023 Sep 14;19(9):e1011430. doi: 10.1371/journal.pcbi.1011430 (PMC10501641; doi:10.1371/journal.pcbi.1011430)
Supplement: S5 Fig — a) Map of variations in offset s, slope α, lapse ∊ and efficiency E, for inference-based agents in the parameter space. The simulations were performed on an ensemble of 15 x 10 inference-based agents with different values of the internal model parameters Prew and Pswitch. b) Example behavior of three inference-based agents taken from the diagonal of the parameter space (dashed line, agents are represented by crosses in panel a plots), illustrating the performance over 100 blocks (top row), and average transition function (bottom row, mean ± standard deviation, n = 1000 blocks). (DOCX) [file pcbi.1011430.s005.docx]

**
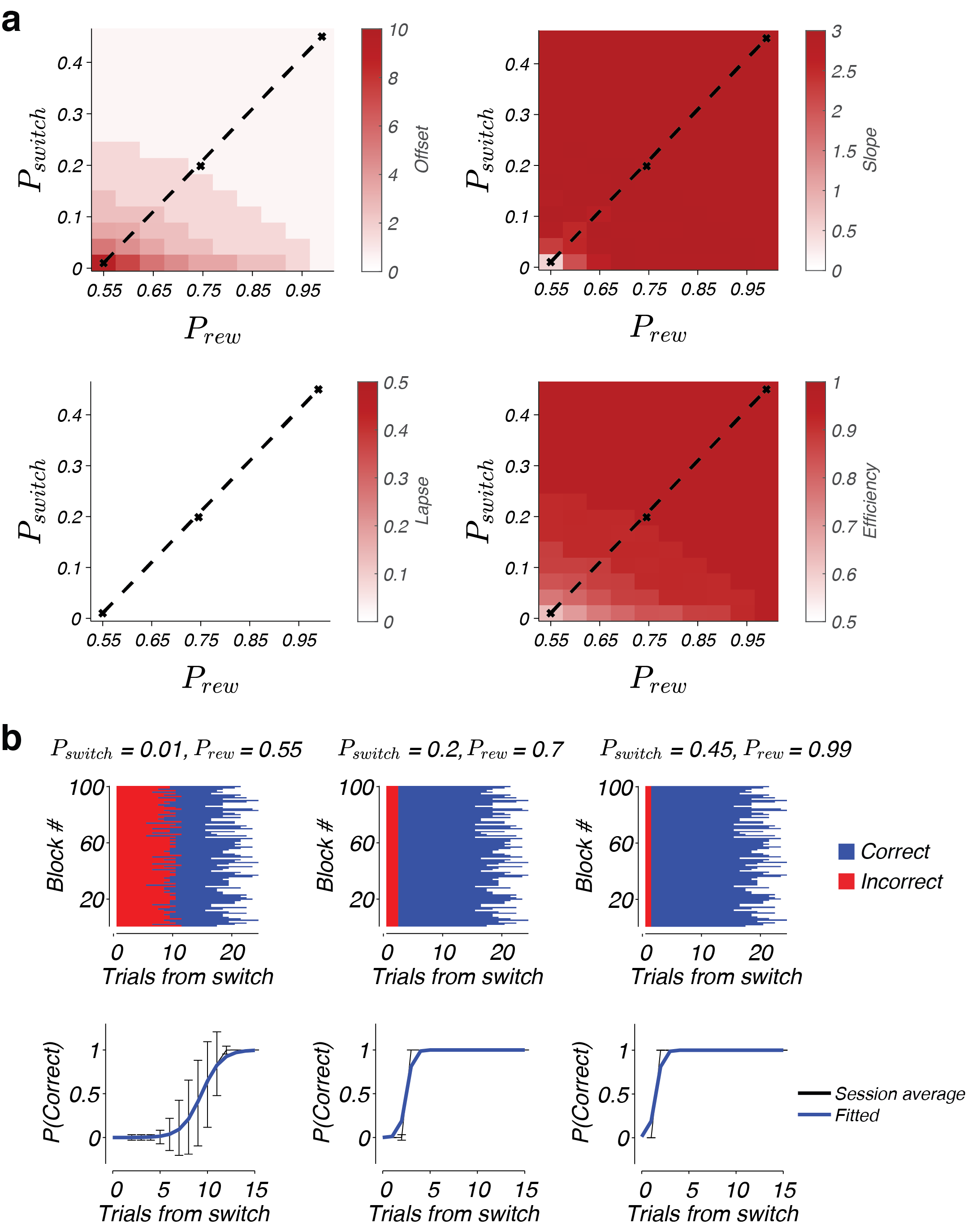
**

**S5 Fig:**  **Variations of four behavioral benchmarks across the inference-based parameter space**. a) Map of variations in offset s, slope 𝛼, lapse 𝜖 and efficiency E, for inference-based agents in the parameter space. The simulations were performed on an ensemble of 15 x 10 inference-based agents with different values of the internal model parameters 𝑃_rew_ and 𝑃_switch_. b) Example behavior of three inference-based agents taken from the diagonal of the parameter space (dashed line, agents are represented by crosses in panel a plots), illustrating the performance over 100 blocks (top row), and average transition function (bottom row, mean ± standard deviation, n = 1000 blocks).
