# Supplementary material for: Mixtures of strategies underlie rodent behavior during reversal learning
Source: PLoS Comput Biol. 2023 Sep 14;19(9):e1011430. doi: 10.1371/journal.pcbi.1011430 (PMC10501641; doi:10.1371/journal.pcbi.1011430)
Supplement: S6 Fig — Approach to segment the model-free and inference-based parameter spaces into distinct behavioral regimes. We performed a computational simulation of an ensemble of Q-learning and inference-based agents taken from grids that spanned the entire two spaces. For each agent, we obtained the transition function and four behavioral features characterizing the sigmoidal fit. We pooled the features of all agents into a feature matrix and applied a density-based approach to cluster these features into six regimes. The regime identities were visualized for all points in the two parameter spaces. After clustering the points, we identified four regimes in the Q-learning space (Q1-4) and two regimes (IB5-6) in the inference-based space. (DOCX) [file pcbi.1011430.s006.docx]

**
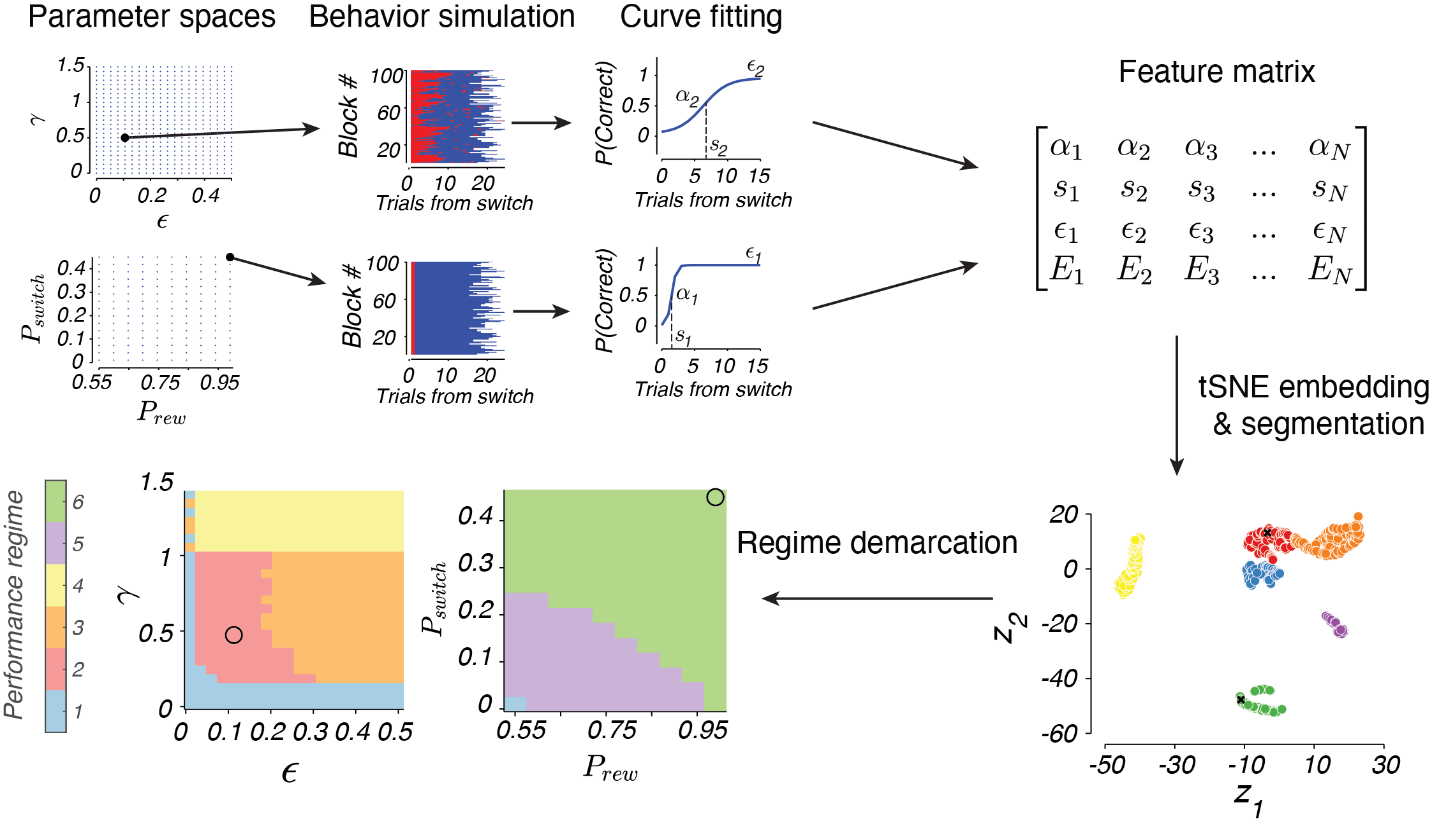
**

**S6 Fig: Subdomains of model-free and inference-based behavior.** Approach to segment the model-free and inference-based parameter spaces into distinct behavioral regimes. We performed a computational simulation of an ensemble of Q-learning and inference-based agents taken from grids that spanned the entire two spaces. For each agent, we obtained the transition function and four behavioral features characterizing the sigmoidal fit. We pooled the features of all agents into a feature matrix and applied a density-based approach to cluster these features into six regimes. The regime identities were visualized for all points in the two parameter spaces. After clustering the points, we identified four regimes in the Q-learning space (Q1-4) and two regimes (IB5-6) in the inference-based space.
