# Supplementary material for: Mixtures of strategies underlie rodent behavior during reversal learning
Source: PLoS Comput Biol. 2023 Sep 14;19(9):e1011430. doi: 10.1371/journal.pcbi.1011430 (PMC10501641; doi:10.1371/journal.pcbi.1011430)
Supplement: S7 Fig — Stacked bar plots in each panel show the composition of the behavioral strategies (Q1-Q4, IB5-IB6) of a single animal over the course of training. Black line on the panel shows the animal’s performance in each session. (DOCX) [file pcbi.1011430.s007.docx]

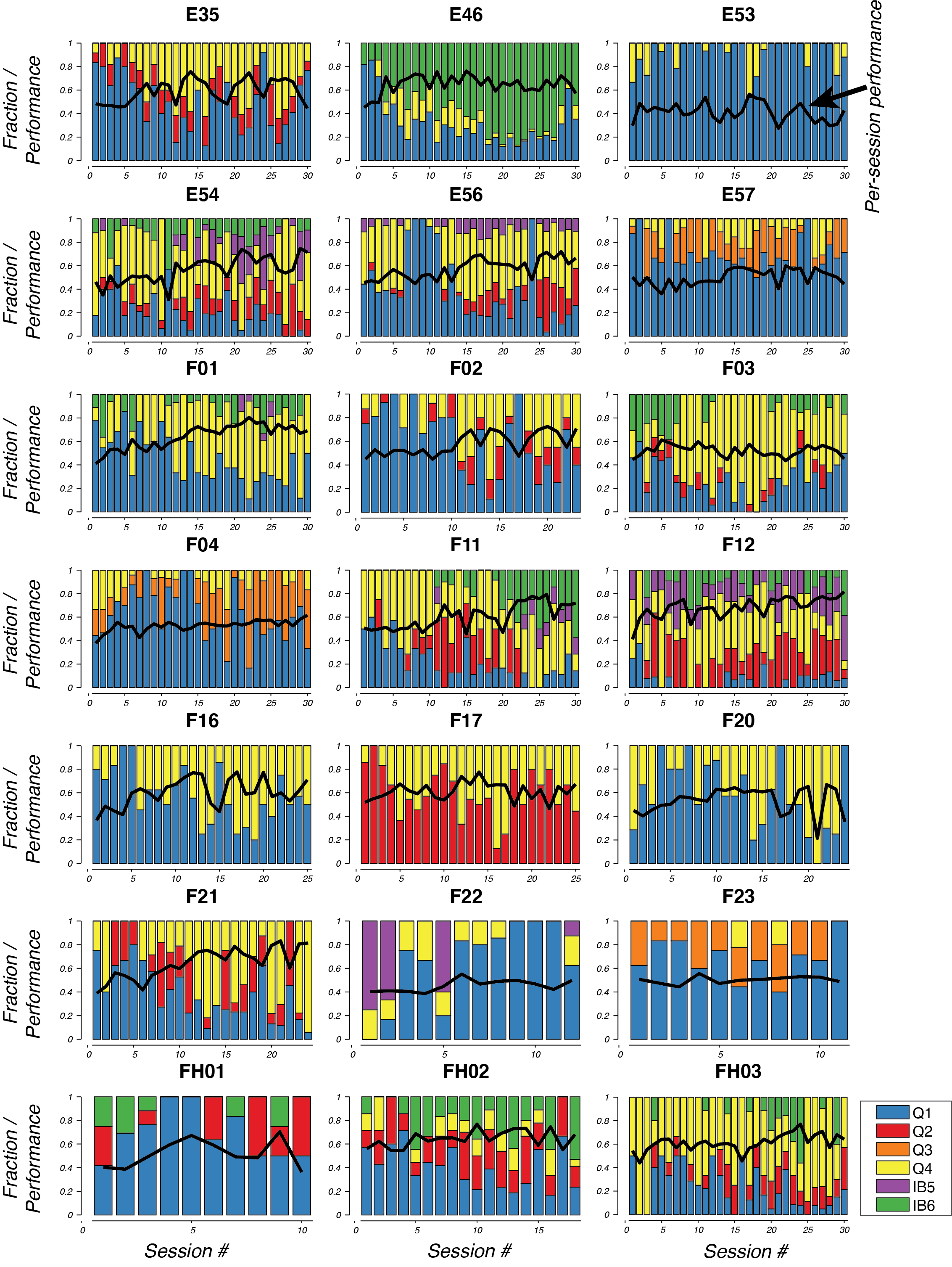


**S7 Fig: Evolution of behavioral strategies of individual mice.** Stacked bar plots in each panel show the composition of the behavioral strategies (Q1-Q4, IB5-IB6) of a single animal over the course of training. Black line on the panel shows the animal’s performance in each session.
