# Supplementary material for: Mixtures of strategies underlie rodent behavior during reversal learning
Source: PLoS Comput Biol. 2023 Sep 14;19(9):e1011430. doi: 10.1371/journal.pcbi.1011430 (PMC10501641; doi:10.1371/journal.pcbi.1011430)
Supplement: S8 Fig — (a) Average frequency (mean ± standard deviation) of decoded blockHMM behavioral strategies (Q1-Q4, IB5-IB6) across training sessions for female (top panel) and male mice (bottom panel). (b) Mean composition of behavioral strategies in male and female mice across three session groups: sessions 1–10 (top), sessions 11–20 (middle), and sessions 21–30 (bottom panel). (n.s: p > 0.05, Mann-Whitney U-test). (DOCX) [file pcbi.1011430.s008.docx]

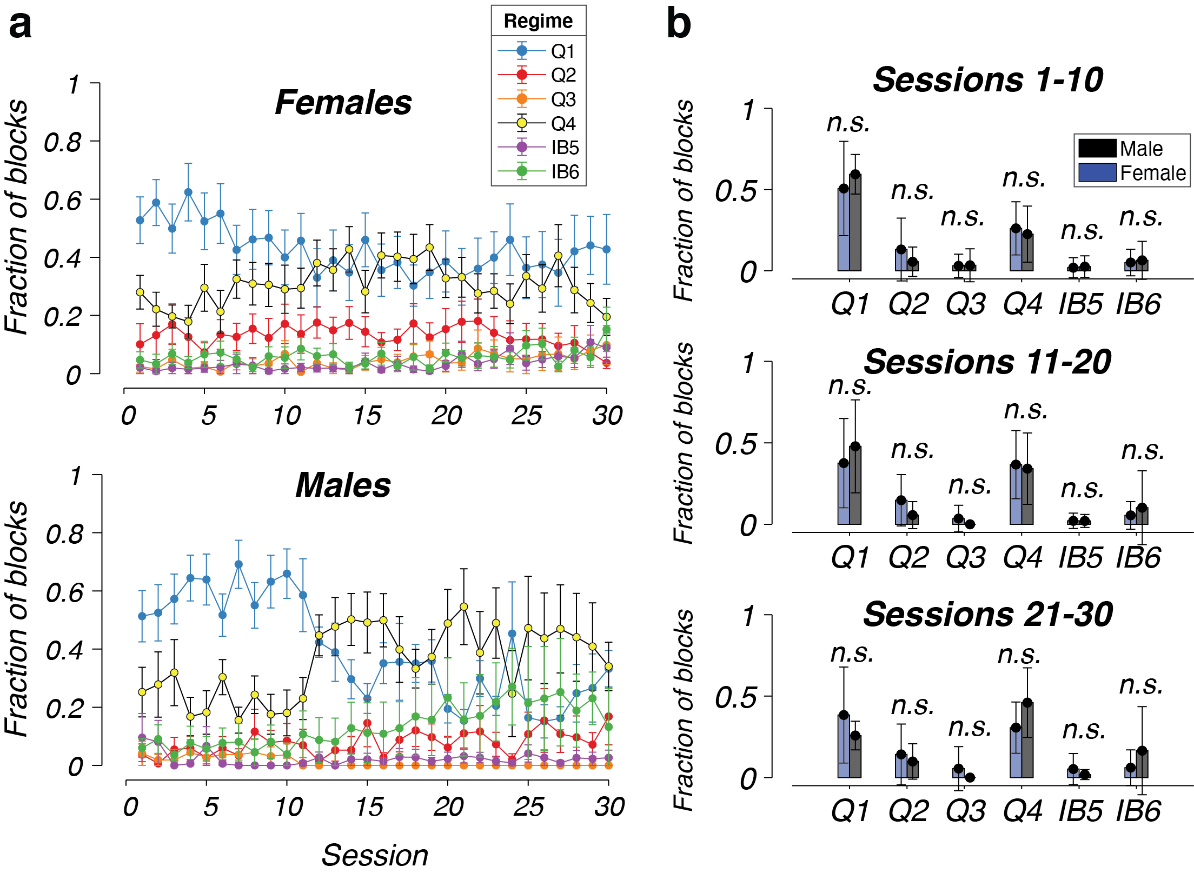


**S8 Fig: Sex differences in composition of blockHMM modes.** (a) Average frequency (mean ± standard deviation) of decoded blockHMM behavioral strategies (Q1-Q4, IB5-IB6) across training sessions for female (top panel) and male mice (bottom panel). (b) Mean composition of behavioral strategies in male and female mice across three session groups: sessions 1-10 (top), sessions 11-20 (middle), and sessions 21-30 (bottom panel). (*n.s*: p > 0.05, Mann-Whitney U-test).
