# Supplementary material for: Mixtures of strategies underlie rodent behavior during reversal learning
Source: PLoS Comput Biol. 2023 Sep 14;19(9):e1011430. doi: 10.1371/journal.pcbi.1011430 (PMC10501641; doi:10.1371/journal.pcbi.1011430)
Supplement: S9 Fig — (a) Distribution of blockHMM mode performances across all experimental animals. BlockHMM modes are classified into low, intermediate and high-performing groups as in Fig 4B. (b) Lapse and offset parameters of individual behavioral modes across all animals. Modes are classified as low-performing (blue), intermediate-performing, high-lapse (yellow), intermediate-performing, high-offset (pink), high-performing (green). A subset of high-performing modes with negative offset are labeled as “Early” switches (black circles). The rest of the high-performing modes are labeled as “Regular” switches. (c) Block transition dynamics of the behavioral regimes identified in (b). Red lines in the bottom panels correspond to the High-performing modes with negative offsets. (d) Frequency of each class of blockHMM modes over the course of training. (DOCX) [file pcbi.1011430.s009.docx]

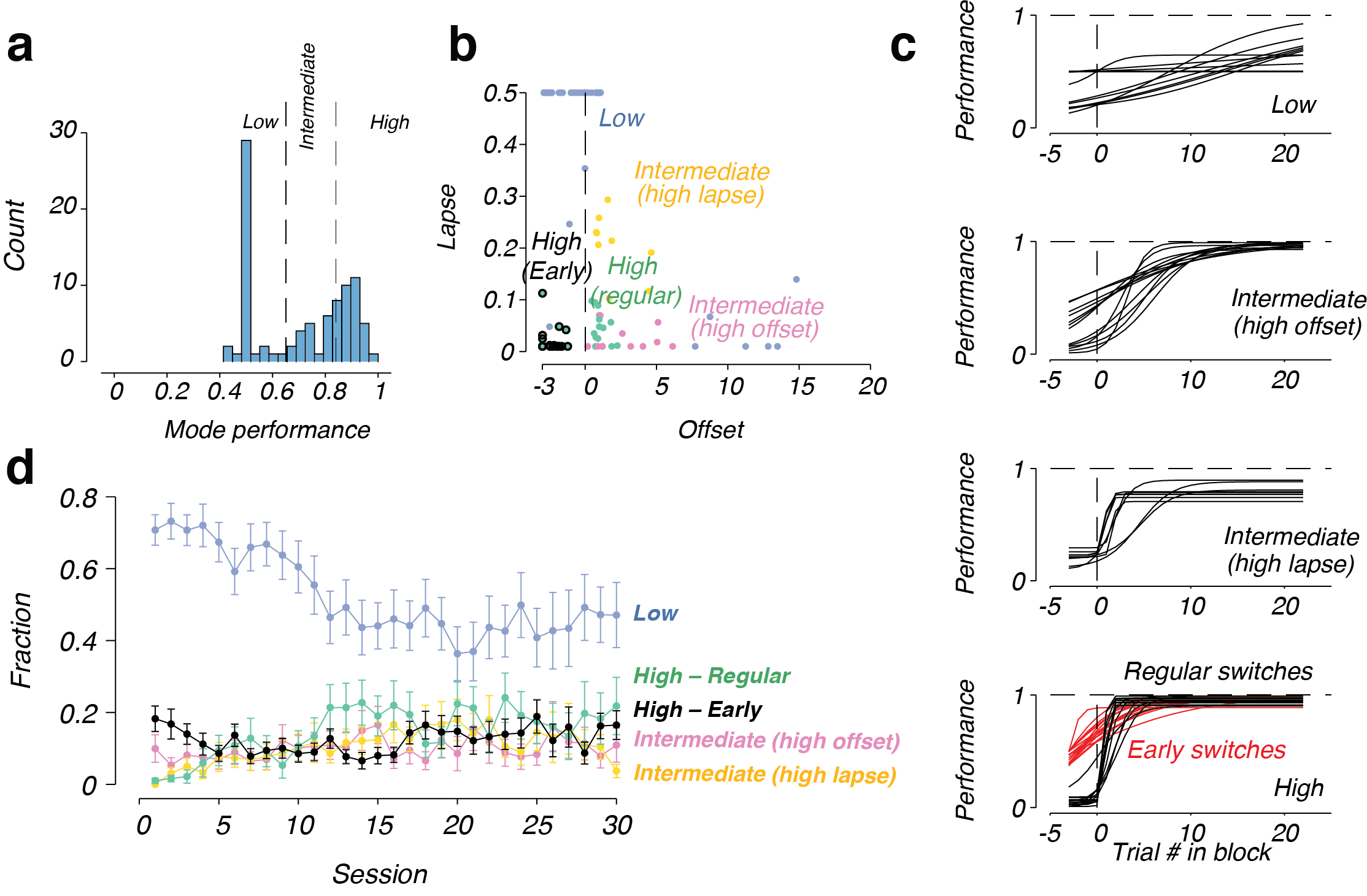


**S9 Fig: Identification of behavioral modes corresponding to early switches.** (a) Distribution of blockHMM mode performances across all experimental animals. BlockHMM modes are classified into low, intermediate and high-performing groups as in Fig 4B. (b) Lapse and offset parameters of individual behavioral modes across all animals. Modes are classified as low-performing (blue), intermediate-performing, high-lapse (yellow), intermediate-performing, high-offset (pink), high-performing (green). A subset of high-performing modes with negative offset are labeled as “Early” switches (black circles). The rest of the high-performing modes are labeled as “Regular” switches. (c) Block transition dynamics of the behavioral regimes identified in (b). Red lines in the bottom panels correspond to the High-performing modes with negative offsets. (d) Frequency of each class of blockHMM modes over the course of training.
