# Supplementary material for: Mixtures of strategies underlie rodent behavior during reversal learning
Source: PLoS Comput Biol. 2023 Sep 14;19(9):e1011430. doi: 10.1371/journal.pcbi.1011430 (PMC10501641; doi:10.1371/journal.pcbi.1011430)
Supplement: Attachment — Submitted filename: PCOMPBIOL-D-23-00531.pdf [file pcbi.1011430.s010.pdf]

Title: A state-space algorithm for dissociating mixtures of strategies during reversal learning

Review comments:

This study by Le et al. provides a much awaited, exciting application of the growing body of state-space modeling approach to the realm of value-based decision making. Specifically, the authors applied a hidden Markov Model at a block level (blockHMM) to uncover the underlying heterogeneous behavioral states (or strategies) in response to reversal in the reward environment. Each of these states is assumed to be governed by three parameters from logistic regression model: the switch offset *s* (latency of switch), the slope or sharpness of choice transition α, and the lapse or error rate $\boldsymbol{\varepsilon}$. While the overall approach is fairly intuitive and straightforward, it leads to an interesting conclusion that the transitional behavior in reversal learning may not be so uniform and could in fact reflect heterogeneity. As authors have pointed out, this bears an important implication for many reversal learning studies, which often assume an abstracted form of uniformity/homogeneity in the observed behavior.

Although the overall performance of the mice suggests that the animals may not have learned to perform the task so well (plateauing around ~65% accuracy, or ~80% if counting only the last 10 trials in each block), the main point that the animals exhibit a mixture of behavioral modes still holds. Therefore I would recommend this article for publication, given that the authors address some of the concerns below:

1. There are several reports that animals come to expect reversals when they are exposed to the same task structure repeatedly over a period of time. For example, Atilgan et al. (2022) has shown that mice come to anticipate reversal and switch their choice prior to the reversal in probabilistic tasks. Similarly, Woo et al. (2023) more recently has shown that mice (as well as monkeys) exhibit expectation of reversals as captured by changes in strategies prior to actual reversal. (Costa et al. (2015), which is already cited in this work, also reports similar results in monkeys.) Given these findings, I think it's worthwhile to consider this as one of the strategies adopted by the animals. If I'm not mistaken, I believe the authors have not considered this as a strategy potentially adopted by (some of) the mice, as the logistic regression assumes that transitory behavior takes place only once the actual reversal has happened (offset parameter $s \geq 0$, #630). Could this parameter (*s*) be actually allowed to have negative values, if we assume that the transition has happened prior to reversals? I think this would be the only way the logistic curve can have a straight line at 1 at trial 0 (which would be observed if mice indeed anticipated reversals). Or if the authors disagree that this is a useful practice, the rationale for it would at least have to be noted (e.g., data shows no such flat lines peaking at 1).
Please see below for the mentioned works above:

- Atilgan, H., Murphy, C. E., Wang, H., Ortega, H. K., Pinto, L., & Kwan, A. C. (2022). Change point estimation by the mouse medial frontal cortex during probabilistic reward learning. *bioRxiv*, 2022-05.
- Woo, J. H., Aguirre, C. G., Bari, B. A., Tsutsui, K. I., Grabenhorst, F., Cohen, J. Y., ... & Soltani, A. (2023). Mechanisms of adjustments to different types of uncertainty in the reward environment across mice and monkeys. *Cognitive, Affective, & Behavioral Neuroscience*, 1-20.

2. One of the main features of Hidden Markov Models is the specification of the transition between hidden states (i.e., transition matrix). However, from the current specification in Methods (Section *BlockHMM fitting to animal behavior*), it is less clear if the analysis of the empirical data takes a full advantage of the proposed HMM framework. That is, in Fig.3 showing simulation results, all trials in the (synthetic) session data are included in the analysis, which also enable inference about transitory dynamics among the hidden states. Yet, when fitting BlockHMM to the animal behavior (Fig.4), it is lacking any inference about the transition relationships between the hidden states. (I was expecting this info since it is relevant to understanding how one behavioral mode is related to another). Is this due to the fact that only the first 15 trials are accounted for by the model? If so, would this be a practical limitation of the model, or just for the ease of analysis (requiring same # of trials across blocks)? Either way, the reason for this should be noted explicitly somewhere, to make it clear that we are looking at the transitory dynamics only and excluding the later trials.

Minor comments:

1. Did the mice show any side biases (toward either Left or Right), that could potentially account for the sub-optimality in the strategy? I was thinking that since the decision is motor-based, as opposed to licking or nose-poking, the choice behavior could be confounded with handedness in mice (Cf. below ref). For example, simply comparing the performance when Leftward block vs. Rightward motion was better could reveal if mice behave differently under each condition.

- Signore, P., Nosten-Bertrand, M., Chaoui, M., Roubertoux, P. L., Marchaland, C., & Perez-Diaz, F. (1991). An assessment of handedness in mice. *Physiology & behavior*, *49*(4), 701-704.

2. For model-free RL, I find the learning rate being greater than 1 somewhat conceptually strange (#376, #747). I see that only the learning rates of > 1 would allow the Regime Q4, but were there other reasons this parameter region was explored? Besides, I'm assuming q_i is bounded to be nonnegative, but please specify whether this is the case in the Methods (#750).

3. For figures that contain data for individual subjects (e.g., Fig.2b, Fig.4e, Fig.6a), I think readers would appreciate if the mean performance/accuracy of each subject is reported next to the individual data, to get an intuition for how each variable is related to the overall performance of individuals. For example, I'm curious whether having more behavioral modes (Fig.4e) is positively/negatively associated with the performance.

4. For Fig.6e, I think it would be also beneficial to have this plot for all individual subjects (as a supplementary figure), for readers to appreciate how the mixture of modes evolve over time. Current Fig.6e is uninformative in that not all animals show all the regimes in the plot shown — it is rather an averaged data across subjects. Perhaps, related to the above comment (#3), the performance plot could be overlaid with the fractions to show how the compositions of modes underlie the performance change.

5. Supp Fig.2b: I assume this is a cumulative distribution across all individuals within each sex, but were there any significant differences between male & female mice in terms of performance

(Fig.1d / Fig.S1) or composition of HMM modes within session (Fig.6e)? With the growing body of work on sex differences in rodent reversal learning (examples below), I think that this study could potentially appeal to wider audiences by noting such differences in the strategies, especially since the work is centered on the heterogeneity of strategies. Thankfully, this experiment has included both male and female mice, which is quite rare to find.

- Chen, C. S., Ebitz, R. B., Bindas, S. R., Redish, A. D., Hayden, B. Y., & Grissom, N. M. (2021). Divergent strategies for learning in males and females. *Current Biology*, *31*(1), 39-50.
    - Male mice exhibiting more volatile/inconsistent strategies compared to females.
- Aarde, S. M., Genner, R. M., Hrncir, H., Arnold, A. P., & Jentsch, J. D. (2021). Sex chromosome complement affects multiple aspects of reversal-learning task performance in mice. *Genes, Brain and Behavior*, *20*(1), e12685.
- Harris, C., Aguirre, C., Kolli, S., Das, K., Izquierdo, A., & Soltani, A. (2021). Unique features of stimulus-based probabilistic reversal learning. *Behavioral neuroscience*, *135*(4), 550.
    - Female rats exhibited longer latencies than males after reversal, but not before reversals.

Other minor edits/typos:

1. The Methods section is missing Equation numbers for all the equations.
2. #653: I think the authors are referring to Fig. 2a, not *5a*.
3. #706: Figure 3c-g also mislabeled as *4c-g*.
4. #769: c_i instead of *c_i*
