# Supplementary material for: Mixtures of strategies underlie rodent behavior during reversal learning
Source: PLoS Comput Biol. 2023 Sep 14;19(9):e1011430. doi: 10.1371/journal.pcbi.1011430 (PMC10501641; doi:10.1371/journal.pcbi.1011430)
Supplement: Attachment — Submitted filename: Le_et_al_2023_Response to reviewers.pdf [file pcbi.1011430.s011.pdf]

We thank the reviewers for their careful reading of our manuscript and for their insightful comments. We have prepared a revised manuscript that carefully addresses all their concerns. In the following, the reviewers' comments are quoted in BLUE, followed by our responses.

Reviewer 1

My only major concerns are:
(1) conclusion 1 above is more or less expected (we know that mouse behavior is often suboptimal in nearly all task settings, and that state-space models can capture these suboptimalities is already published). Relatedly, the adaptation of these previously published state-space models here seems quite modest. The authors may want to temper/qualify (or perhaps, fortify/better justify, if authors disagree) claims of novelty around the HMM mixture model itself (i.e. in the abstract, title, less emphasis on the state-space model versus the conculsions drawn for it in combination with the model agent simulations)

We agree with reviewer 1 that available evidence points to mouse behavior being suboptimal in most task settings, and that state-space models have been developed for other task contexts. However, specifically in the study of reversal learning, a vast majority of studies have assumed that mice execute a uniform strategy, and state-space models have not been used to characterize the mixture of strategies to further quantify the suboptimality in their behavior. Thus, blockHMM fills this gap by allowing the investigation of block-by-block shifts in behavioral strategies, which further clarifies the sources of sub-optimal behavior of mice throughout the task.

We thank the reviewer for suggesting ways we can contextualize our findings and make the findings more relevant. We have further clarified the claims of novelty around the HMM mixture model to make clear the relation to previous HMM models that were previously developed (Introduction: lines 125-128). We have changed the title of the paper to highlight the analysis of the mixtures of strategies, rather than the development of the blockHMM itself. We have increased the emphasis on additional findings of the model (around sex differences and the characterization of early switching) and added more context in the discussion to address this concern (lines 531-535; 559-564).

(2) Relatedly, the adapted state-space model here seems of somewhat limited utility outside of this study. I imagine the utility of a new model should be a) it's ability to describe behavior in new depth (i think the authors adequately meet this bar, although caveat for point 1 above at the high level), and b) providing new parameters for linking reward-based decisions with their underlying neural correlates. Because the model here describes decisions in blocks of choices, it seems unlikely to be very useful for the latter (perhaps it provides a toehold into the neural basis of block transitions?). In addition, this model is only suitable to describe reversal learning tasks with clear block structures. My main suggestion here: can the authors apply their blockHMM to a seperate dataset from a similar related revesal learning task (perhaps datasets from the Intl Brain Lab?). I imagine this could support their main conclusions and demonstrate a broader utility of the model. If this is not possible or feasible, I would ask to better fortify the more general utility of this modeling framework (compared to prior efforts) with regards to a) understanding the neural basis of mixed strategies and b) behavior in related but distinct contexts.

Thank you for the suggestions on how to broaden the utility of the model. While we acknowledge that the blockHMM is useful in specific experimental settings that involve blocks of trials, we also think that the model has a very broad general utility in answering various research questions in reward-guided decision-making and beyond.

In terms of probing neural correlates underlying reversal learning, the states decoded by the model will offer valuable information into the decoding of state-dependent neural correlates involved in different states of decision making. We have shown through our simulations that the states inferred by our model can be mapped on to model free and inference based behavioral modes, which involve vastly different brain regions and networks. The mixture of strategies that we revealed through this study presents an interesting possibility that these brain regions might display very dynamic encoding in different blocks of trials during single behavioral sessions. examine the encoding of choice, reward and switching in brain-wide and single-neuron responses in these different states which are inferred by the block Hidden Markov Model. We clarified these points in a more extended section in the Discussion section (lines 581-588).

About demonstrating the utility of our model in related contexts, we unfortunately were not able to find a dataset involving reversal learning from the International Brain Lab data as the existing datasets mainly involve visually-guided decision-making tasks with no reversal in reward-contingency. However, we believe the model can be used to study similar decision-making tasks where different sets of task rules are applied in a block-wise fashion. We have also included this in the Discussion section, referencing example tasks that will potentially benefit from our approach (lines 588-590).

Minor points/questions:

(1) Fig 3e-- can the authors add the actual values of state transitions rather than colorscale to better evaluate
We have added the numerical values of the state transitions for easier comparison in Fig.3e (line 248).

(2) Fig4 -- is the blockHMM fit to each individual animals or concatenaged data? I would be curious to see the cross-validated log-likliehood for different numbers of states in the data (related to Supp Fig 2a), as well as the transition matrices for individual mice or the group.

The blockHMM is fit to individual animals. We have added the following plots to better demonstrate the individual animal fitting: the cross-validated log-likelihood for different numbers of states per animal (Supp. Fig. 2a; line 1101), and the transition matrices for individual mice (Supp Fig. 3; line 1110).

Reviewer 2:

There are several reports that animals come to expect reversals when they are exposed to the same task structure repeatedly over a period of time. For example, Atilgan et al. (2022) has shown that mice come to anticipate reversal and switch their choice prior to the reversal in probabilistic tasks. Similarly, Woo et al. (2023) more recently has shown that mice (as well as monkeys) exhibit expectation of reversals as captured by changes in strategies prior to actual

reversal. (Costa et al. (2015), which is already cited in this work, also reports similar results in monkeys.) Given these findings, I think it's worthwhile to consider this as one of the strategies adopted by the animals. If I'm not mistaken, I believe the authors have not considered this as a strategy potentially adopted by (some of) the mice, as the logistic regression assumes that transitory behavior takes place only once the actual reversal has happened (offset parameter s ≥ 0, #630). Could this parameter (s) be actually allowed to have negative values, if we assume that the transition has happened prior to reversals? I think this would be the only way the logistic curve can have a straight line at 1 at trial 0 (which would be observed if mice indeed anticipated reversals). Or if the authors disagree that this is a useful practice, the rationale for it would at least have to be noted (e.g., data shows no such flat lines peaking at 1).

Thank you for this insightful comment. We have updated our fitting procedure to consider the possibility that animals might anticipate the reversal and switch their actions prior to the reversal in certain blocks. In our new analysis setup (which we include as a supplementary analysis in Supp. Fig. 9; line 1158), we included the animals' choices three trials prior to each block switch in addition to the trials within the block. This effectively relaxes our constraint to $s > -3$. This choice leaves the fitting tractable while allowing us to investigate the possibility of early switching.

Interestingly, we found that there are indeed switching modes that correspond to negative offsets (Supp. Fig. 9a-c), suggesting that animals indeed make switches prior to the actual reversal in these blocks. The frequency of these early switches remains stable throughout training (Supp. Fig. 9d). The identification of these early switches with blockHMM should help future investigations into neural correlates of anticipatory switching or early switching due to exploration. We have added these results and discussion to the manuscript.

2. One of the main features of Hidden Markov Models is the specification of the transition between hidden states (i.e., transition matrix). However, from the current specification in Methods (Section BlockHMM fitting to animal behavior), it is less clear if the analysis of the empirical data takes a full advantage of the proposed HMM framework. That is, in Fig.3 showing simulation results, all trials in the (synthetic) session data are included in the analysis, which also enable inference about transitory dynamics among the hidden states. Yet, when fitting BlockHMM to the animal behavior (Fig.4), it is lacking any inference about the transition relationships between the hidden states. (I was expecting this info since it is relevant to understanding how one behavioral mode is related to another). Is this due to the fact that only the first 15 trials are accounted for by the model? If so, would this be a practical limitation of the model, or just for the ease of analysis (requiring same # of trials across blocks)? Either way, the reason for this should be noted explicitly somewhere, to make it clear that we are looking at the transitory dynamics only and excluding the later trials.

The block HMM indeed provides information about transitory relationships between states through the inferred transition matrices for each animal, encapsulated in the transition matrix T. We have added this information in supplementary figure 3 (line 1110).

We chose to include the first 15 trials of each block for several reasons. First, this equalizes the block lengths across all blocks and provides a rectangular data matrix to be used for blockHMM fitting. Second, it avoids the model overfitting to the particularly long blocks, which often occurs when the performance is poor (in our protocol, block switches only occur when the rolling average reaches a threshold of 0.7). The assumption with truncating blocks to the first 15 trials

is that the transitory dynamics of the block has stabilized by trial 15. This assumption is justified by the fact that the majority of our modes has stabilized by trial 10 in the block (Fig. 4d).

Minor comments

1. Did the mice show any side biases (toward either Left or Right), that could potentially account for the sub-optimality in the strategy? I was thinking that since the decision is motor-based, as opposed to licking or nose-poking, the choice behavior could be confounded with handedness in mice (Cf. below ref). For example, simply comparing the performance when Leftward block vs. Rightward motion was better could reveal if mice behave differently under each condition.
● Signore, P., Nosten-Bertrand, M., Chaoui, M., Roubertoux, P. L., Marchaland, C., & Perez-Diaz, F. (1991). An assessment of handedness in mice. Physiology & behavior, 49(4), 701-704.

This is an interesting suggestion. We have investigated the difference in performance between left and right blocks and did not find any significant side biases on average (Supp. Fig. 1c; line 1086), although we found biases in individual animals (Supp. Fig. 1d). In addition, the magnitude of the side bias does not seem to correlate with overall performance (Supp. Fig. 1d).

2. For model-free RL, I find the learning rate being greater than 1 somewhat conceptually strange (#376, #747). I see that only the learning rates of > 1 would allow the Regime Q4, but were there other reasons this parameter region was explored? Besides, I'm assuming q_i is bounded to be nonnegative, but please specify whether this is the case in the Methods (#750).

In general situations with arbitrary number of states, values, and trial-by-trial state transitions, learning rates greater than 1 indeed are rarely used since they might lead to instabilities and divergence in the agent's learned values. However, in our case, because of the block-wise and structured nature of the task, where only two values are seen by the agents and the two values alternate block by block, learning rates in this regime do not lead to instability. For this same reason, there was also no need to bound the q_i to be non-negative. Thus, we decided to explore the regime since it is still a valid parameter range that does not lead to unreasonable behavior of the agents. Since the behavior in this regime was also qualitatively distinct from the other Q-learning regimes (Fig. 6b), we believe our investigation of the regime was justified.

3. For figures that contain data for individual subjects (e.g., Fig.2b, Fig.4e, Fig.6a), I think readers would appreciate if the mean performance/accuracy of each subject is reported next to the individual data, to get an intuition for how each variable is related to the overall performance of individuals. For example, I'm curious whether having more behavioral modes (Fig.4e) is positively/negatively associated with the performance.

Thank you for the suggestion. We have added this information when possible. In Fig. 2b (line 214), we included the performance next to the box plots. We included an additional plot in Supp. Fig. 1c (line 1086) showing the relation between the number of modes and performance (supplementary to Fig. 4e and 6a). In Fig. 6c-d (line 456) we have included the average performance of the two animals on the plots.

4. For Fig.6e, I think it would be also beneficial to have this plot for all individual subjects (as a supplementary figure), for readers to appreciate how the mixture of modes evolve over time. Current Fig.6e is uninformative in that not all animals show all the regimes in the plot shown — it is rather an averaged data across subjects. Perhaps, related to the above comment (#3), the

performance plot could be overlaid with the fractions to show how the compositions of modes underlie the performance change.

We have included an additional plot in Supp. Fig. 7 (line 1148) showing the per-session performance of each animal overlaid on top of the evolution of the mixture compositions (supplementary to Fig. 6e).

5. Supp Fig.2b: I assume this is a cumulative distribution across all individuals within each sex, but were there any significant differences between male & female mice in terms of performance (Fig.1d / Fig.S1) or composition of HMM modes within session (Fig.6e)? With the growing body of work on sex differences in rodent reversal learning (examples below), I think that this study could potentially appeal to wider audiences by noting such differences in the strategies, especially since the work is centered on the heterogeneity of strategies. Thankfully, this experiment has included both male and female mice, which is quite rare to find.

These are very important suggestions to add value to our approach and help dissect sex differences in rodent reversal learning. To add on to our investigation of sex differences in mixtures of strategies, we have included Supp. Fig. 1e,f (line 1086) and Supp. Fig. 8 (line 1152) to show the result of these additional analyses.

In terms of performance, we found that males on average had higher performance than females in the later sessions of training (sessions 21 - 30). However, the difference was not statistically significant, as animal-to-animal variability was high (Supp. Fig. 1e,f). Similarly, we observed small differences in the average fractions of the blockHMM modes between males and females, but these differences were again not statistically significant (Supp. Fig. 8). Our study was not particularly powered to distinguish the differences in strategies between sexes, but this hypothesis could be further explored in future studies.

Other minor edits/typos:

1. The Methods section is missing Equation numbers for all the equations.
Done (lines 354, 686, 739, 745, 749, 764, 772, 787, 813, 822-825, 838-839).

2. #653: I think the authors are referring to Fig. 2a, not 5a.
Done (line 714).

3. #706: Figure 3c-g also mislabeled as 4c-g.
Done (line 768).

4. #769: ci instead of c_i
Done (line 827).
